# Gaze direction as a facial cue of memory retrieval state

**DOI:** 10.3389/fpsyg.2022.1063228

**Published:** 2022-12-22

**Authors:** Anaïs Servais, Christophe Hurter, Emmanuel J. Barbeau

**Affiliations:** ^1^Centre de Recherche Cerveau et Cognition (CerCo), CNRS-UPS, UMR5549, Toulouse, France; ^2^Ecole Nationale d’Aviation Civile (ENAC), Toulouse, France

**Keywords:** gaze aversion, autobiographical memory retrieval, eye movements, facial cues, social cues, internal attention

## Abstract

Gaze direction is a powerful social cue that indicates the direction of attention and can be used to decode others’ mental states. When an individual looks at an external object, inferring where their attention is focused from their gaze direction is easy. But when people are immersed in memories, their attention is oriented towards their inner world. Is there any specific gaze direction in this situation, and if so, which one? While trying to remember, a common behavior is gaze aversion, which has mostly been reported as an upward-directed gaze. Our primary aim was to evaluate whether gaze direction plays a role in the inference of the orientation of attention—i.e., external vs. internal—in particular, whether an upward direction is considered as an indicator of attention towards the internal world. Our secondary objective was to explore whether different gaze directions are consistently attributed to different types of internal mental states and, more specifically, memory states (autobiographical or semantic memory retrieval, or working memory). Gaze aversion is assumed to play a role in perceptual decoupling, which is supposed to support internal attention. We therefore also tested whether internal attention was associated with high gaze eccentricity because the mismatch between head and eye direction alters visual acuity. We conducted two large-sample (160–163 participants) online experiments. Participants were asked to choose which mental state—among different internal and external attentional states—they would attribute to faces with gazes oriented in different directions. Participants significantly associated internal attention with an upward-averted gaze across experiments, while external attention was mostly associated with a gaze remaining on the horizontal axis. This shows that gaze direction is robustly used by observers to infer others’ mental states. Unexpectedly, internal attentional states were not more associated with gaze eccentricity at high (30°) than low (10°) eccentricity and we found that autobiographical memory retrieval, but not the other memory states, was highly associated with 10° downward gaze. This reveals the possible existence of different types of gaze aversion for different types of memories and opens new perspectives.

## 1. Introduction

Gaze *direction* is a powerful social cue that allows people to decode others’ mental states (Baron-Cohen et al., 2001), as it indicates the direction of attention (Droulers and Adil, 2015). People also interact (Emery, 2000) with eye contact or eye-to-face (Pasupathi, 2001; Turkstra, 2005). If the speaker looks away, the listener’s gaze automatically follows so that attention is directed toward the same location (Nummenmaa and Calder, 2009)—a form of *joint attention* (Frischen et al., 2007) in which the gaze is used to detect another person’s focus of attention, in particular when directed toward an object or an event in the external environment. Taxonomies of attention differentiate external from internal attention—this latter being oriented toward internally generated information, such as memories or thoughts (Chun et al., 2011). Internal attention plays an important role during social interactions. In everyday conversations, people spend much time discussing autobiographical memories (Beike et al., 2017) because of their social functions, such as the development or maintenance of intimate relationships (Alea and Bluck, 2003). During a conversation, individuals can engage in *joint reminiscing* when they remember a past event that they have experienced together (Hoerl and McCormack, 2005). People also tend to share their personal memories with others who were not present at the time of the event—so-called *Me memories* (Beike et al., 2017).

When the attention of the speaker is focused on their internal mental world, the listener has to understand that the speaker’s attention is not focusing on external stimuli and that it is, therefore, useless to look at the same location as the speaker. A recent study has shown that people are able to identify whether the attentional focus of others is internally or externally oriented (Benedek et al., 2018). The authors first recorded videos of actors while they were doing either an external task with attention focused on the screen or an internal task involving imagination. Then those videos were presented to a large sample of participants during an online survey asking them to evaluate the mental state of the actor on a continuous slider scale ranging from “attention is clearly internally directed” to “attention is clearly externally directed.” In a first condition, participants were presented the video clips. In the second, they were only presented screenshots taken from the video clips. In a third condition, they were presented the same screenshots with a mask hiding the region of the eyes. Results showed that participants performed better at the task when the region of the eyes was visible, which is consistent with the fact that internal attention has been associated with different types of eye movements (e.g., Benedek et al., 2017; Walcher et al., 2017). However, Benedek et al. (2018) did not report what were the objective signs or what particular features of the gaze people used to make their judgments.

*Gaze direction* could be a potential facial cue of an internal state. This idea is supported by previous studies showing that children can infer that a person is thinking when the person is looking away (i.e., internal attention; Baron-Cohen and Cross, 1992) and that humans tend to attribute thoughtfulness to virtual agents when the agents avert their gaze (Admoni and Scassellati, 2017). This is congruent with the fact that a common behavior, easily observable in daily life, is to shift the gaze away when answering memory questions—a behavior called *gaze aversion* (Glenberg et al., 1998). According to *the cognitive load hypothesis of gaze aversion* (Abeles and Yuval-greenberg, 2017), gaze aversion brings the eyes away from the external distractors present in the surrounding environment to optimize the performance of tasks requiring internal attention. However, Baron-Cohen and Cross (1992) used only stimuli where the gaze was averted to the upward part of the space and linked it to the general term “*thinking.*” Therefore, little is known about the direction of the gaze and whether different directions are interpreted by observers as indicating different internal attentional states.

Even though gaze aversion during memory retrieval has been described as searching for the answer *on the ceiling* or *in the sky* (Doherty-Sneddon and Phelps, 2005) or even referred to as *head in the clouds* (Previc et al., 2005), suggesting that the gaze is directed upward during memory retrieval, such behavior has in fact mostly been interpreted as looking at neutral parts of the surrounding space, containing fewer visual distractors (Salvi and Bowden, 2016). Overall, gaze aversion has received little attention, and to the best of our knowledge, no scientific study has evaluated if specific directions of the gaze could be interpreted by observers as being related to specific mental states. Quite strangely, this topic has mostly been addressed by pseudoscience. For example, synergology or neuro-linguistic programming (NLP) have tried to make specific associations between gaze directions and internal cognitive processes. The Eye-Accessing Cues (EAC) model of NLP conveys the idea that the direction of non-visual eye movements appearing during internal cognition indicates the sensory system involved in the representation the person has in mind: for example, memory retrieval would be associated with a gaze looking up to the left (Wiseman et al., 2012). Recent reviews, however, showed that the majority of the studies trying to replicate the postulates of the EAC model did not support it (Diamantopoulos et al., 2009; Rochat et al., 2018). It is therefore a problem if the general public, not least scientists, have to refer to such pseudoscience when looking for the interpretation of gaze direction during social interactions.

In this study, our primary aim was to evaluate whether gaze direction plays a role in the inference of the orientation of attention, i.e., external vs. internal—in particular, whether an upward direction was indicative of attention directed towards the internal world. Our secondary objective aimed at exploring whether different gaze directions were consistently attributed to different types of internal mental states, and more specifically memory states. Although this part of the study was exploratory, we expected to find differences since different types of memory retrieval involve different phenomenological experiences and states of consciousness (e.g., see Tulving, 2002; Renoult et al., 2019 for the distinction between episodic and semantic memory).

**Figure 1:**
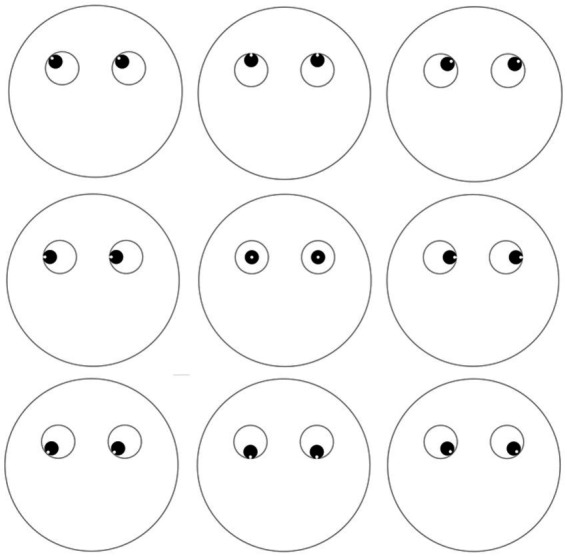
Stimuli presented in Experiments 1a and 1b.

We conducted two online experiments. In the first part of experiment 1 (experiment 1a), we used simplified face stimuli (Figure 1) with eyes oriented in different directions. Gaze direction was set to different coordinates. For each gaze direction, participants had to choose which mental state matched the best among several proposed options. The options, descriptive phrases or statements, were grouped into three categories (participants being unaware of these): external attention, internal attention, and control mental states that were not related to a specific attentional state. To explore the differences between different types of internal attention, this category contained semantic retrieval, autobiographical retrieval, and working memory state. The main hypothesis was that people would associate internal attention with gaze aversion (i.e., gaze directed away). This hypothesis is derived from the *cognitive load hypothesis* (Abeles and Yuval-Greenberg, 2017), which suggests that gaze aversion brings the eyes away from the external distractors present in the surrounding environment to optimize the performance of tasks requiring internal attention. We, therefore, expected internal attention to be more often associated with upward-directed gaze, since gaze aversions during conversations are mostly directed upward, in particular during cognitive effort (Andrist et al., 2014). Moreover, as previously mentioned, gaze aversion has been described as searching for an answer *on the ceiling* or *in the sky* (Glenberg et al., 1998) or even referred to as *head in the clouds* (Previc et al., 2005)—probably because those are neutral spaces with few visual distractors.

The second part of experiment 1 (experiment 1b) aimed to investigate the robustness of this effect. Participants performed the reverse task: where they were asked to associate a gaze direction to a given mental state. They were presented with written sentences describing specific mental states and were asked to choose which of several faces with different gaze directions was associated with the state described.

In a second experiment, we used 3D models of faces, which allowed us to modulate the eccentricity of the gaze—i.e., how far the eyes would rotate. At greater eccentricity (>20° rotation from the central primary position), the eye position is less stable and visual acuity is altered (Land and Tatler, 2009; Nakashima and Shioiri, 2014). Because the putative role of gaze aversion is assumed to decrease visual processing to optimize internal attention (Abeles and Yuval-greenberg, 2017), we expected people to associate internal attention with high-eccentricity more than low-eccentricity gazes. In this second experiment, we aimed to test this hypothesis by comparing two levels of eccentricity: approximately 10° and 30°. Above 20°, head movements are generally required to keep the eyes in a comfortable oculomotor range (Land and Tatler, 2009; Nakashima and Shioiri, 2014). The 10° and 30° values were therefore chosen to make sure that one is sufficiently above and the other sufficiently below the threshold of 20°.

## 2. Materials and methods

This study was approved by the Ethics Committee for Research of the University of Toulouse (agreement 2020–273). It is based on two experiments that have been conducted online. Experiments 1a and 1b were conducted with the same sample and Experiment 2 with a separate sample. Sample sizes were based on *a priori* power analyses using G*Power (Version 3.1.9.2; Faul et al., 2009). To achieve sufficient statistical power (>0.80) to detect medium-sized effect (w = 0.30) with alpha.05 on the chi-square test (χ^2^), a sample of at least 160 participants was needed for each experiment.

The experiments were implemented on a Limesurvey server hosted by in our lab (CerCo, Toulouse, France). Participants completed the experiment on their laptop, digital tablet, or smartphone. Participants were recruited through social networks, mailing lists, and the diffusion of flyers with QR codes. Upon clicking on the link to the questionnaire, participants were presented with one page of information about the purpose of the study, which they were asked to read. They were then invited to tick inclusion criteria to ensure that they were between 18 and 50 years old, were French speakers, and did not have psychiatric or neurologic history. To ensure that participants completed only one questionnaire (either Experiments 1a and 1b or Experiment 2), we also required them to tick a box to confirm that they were not involved in another similar study. If one or more criteria were not satisfied, participants were directed toward a “thank you” page. If all the criteria were met, participants were asked to agree to a consent form electronically. Instructions were presented to the participants who were then invited to complete the survey. The specific design of each experiment is described in the related sections (a screenshot of a trial as displayed for the participant is available in Supplementary Figure 1). To ensure that participants read the questions and were genuinely trying to do the experiment, we inserted a validation trial among the real trials in each experiment: they were explicitly asked to choose a specific irrelevant proposition (e.g., “For this question, choose the proposition saying that you like strawberries”). If participants gave an incorrect response on the validation trial, all of their responses were discarded. After completing the survey, demographic data were gathered (age, gender, laterality, level of study, profession). All the collected data were strictly anonymous. The questionnaires were designed to take approximately 15–20 min. In conformity with ethics rules, participants were allowed to disconnect and erase their responses at any time during the study.

### 2.1. Experiment 1a

#### 2.1.1. Participants

Data were collected in June and July 2020. A total of 329 participants clicked on the link to start the study; 180 participants completed the study until the end, but 20 gave a wrong answer to the validation trial. The results thus include responses from 160 participants (60% women; 82.5% right-handed) aged 18–50 years (*M* = 29.28 ± 8.45), with an education level between 5 and 20 years (*M* = 15.86 ± 2.84).

#### 2.1.2. Materials and survey

Participants were presented drawings representing faces (Figure 1). We used simple drawings as stimuli rather than images of real faces in order to be able to control gaze direction angle as a parameter and to avoid interference with other facial features. Faces differed depending on the orientation of the eyes in nine specific directions, defined as follows, according to the position of eyes relative to the face in polar coordinates: 0° (leftward gaze), 45° (up-left gaze), 90° (straight-up gaze), 135° (up-right gaze), 180° (rightward gaze), 225° (down-right gaze), 270° (straight-down gaze), 315° (down-left gaze), and central primary position (straight-ahead gaze). Each face was accompanied by a multiple-choice question where participants were asked to choose, among several descriptions, the one that best corresponded to the mental state represented by the face. For each face, participants had the choice between the same 8 options. The options were related to 3 internal attentional states (autobiographical memory, semantic memory, working memory), and 3 external attentional states (top-down selective attention, bottom-up attention, vigilance) described in the taxonomy of attention (Chun et al., 2011) as well as 2 control options (shyness, lying). Details about the options are reported in Table 1 (descriptions were presented in French but have been translated into English for this article). The participants were simply asked to choose one of the eight mental state option and were not aware of their categorization in three subgroups. Here is the English translation of the instructions provided to the participants: “*You will see drawings of faces. For each face, you have to choose, among several proposals, the one that you think corresponds best to it. There is no right or wrong answer, you have to rely on your impression and choose only one proposal. If you do not know, please select ‘No answer’. This is scientific research. It is therefore important that you read the questions correctly and answer them as seriously as possible so as not to bias the results.”* We proposed the following scenario to help the participants: “*Imagine that the stimulus is a person in front of you. How would you interpret their mental state?*” For all the questions, participants had the option of ticking “I do not know” if they were unsure and preferred to not answer. Each face was presented twice in order to measure intra-individual concordance. Experiment 1a was therefore composed of 18 experimental trials plus a validation trial (*“This trial will allow us to make sure that you completed the survey seriously. For this trial, please choose the statement* ‘It is sunny today’.*”*). Trials were presented one by one in randomized order. For every trial, the eight mental state options were presented in a different random order.

**Table 1:** Mental states categories and corresponding descriptive labels provided to participants.

Cognition type	Cognitive state	Description
Internal attention	Autobiographical memory	Thinking of personal memories (e.g., an event lived in the past).
	Semantic memory	Thinking of general knowledge (e.g., famous event or person).
	Working memory	Trying to memorize a phone number.
External attention	Vigilance	Watching something while being on the lookout.
	Selective top-down attention	Looking for something (e.g., an object).
	Bottom-up attention	Being surprised by an event that catches attention.
Control	Lying	Lying.
	Shyness	Being uncomfortable and avoiding eye contact.

Please note that the information displayed in the “Cognition type” and “Cognitive state” columns were not available to participants, who only saw the content of the “Description” column (refer to the Figure in Supplementary material for the exact material). Note also that the order of presentation of the 8 labels (or 9 if we consider the « no answer » option) was randomized for each trial.

Instructions emphasized that there was no right or wrong answer. Participants were encouraged to trust their first impression in choosing their answer. They were told to not answer at random if they did not know the answer but rather to choose the option “I do not know.” All the stimuli needed to reproduce the experiment are available on OSF.^1^ At the end of the experiment, participants had the possibility to leave free commentaries about the experiment.

#### 2.1.3. Analyses

Raw data were exported as an Excel file (publicly available on OSF). Analyses were performed with Python 3.7.6 and TIBCO Statistica 13.5.0.17. To evaluate the intra-individual consistency of responses, we calculated the Cohen’s Kappa coefficient on responses given by participants for the two presentations of the same gaze direction during Experiment 1a. This coefficient was calculated for each of the nine gaze directions separately. Values were interpreted according to the criteria proposed by Landis and Koch (1977) where coefficient < 0.20 indicates a slight agreement, values between 0.21 and 0.40 are interpreted as fair consistency, values between 0.41 and 0.60 as moderate consistency, and values above.61 as substantial to strong consistency.

To evaluate whether people preferentially associated certain mental states with specific gaze directions, we compared the frequencies at which participants from our sample chose a specific mental state for each gaze direction by means of Pearson’s chi-square test (χ^2^), testing the independence of variables (mental state vs. gaze direction) in a contingency table. In case of significant results, we planned to investigate if mental states could be predicted from gaze directions with probabilities above chance level. We therefore ran a multinomial logistic regression with the responses of participants (mental state choices) as dependent categorical variable and gaze direction of the stimuli as independent categorical variable. We calculated both the overall accuracy of the model and the probability estimates for every gaze direction.

We expected internal attention to be associated with upward gazes (up left, up right, or straight up), external attention to be associated with gaze on the horizontal line (leftward, rightward, or straight ahead), and lying and being shy with downward gazes (straight down, down right, or down left). To test whether the three groups of cognitive states that we had conceived made sense, we ran k-means cluster analyses with three clusters, and we expected the mental states to be grouped based on the frequencies at which they were associated with gaze directions.

#### 2.1.4. Results

Participants abstained from answering on an average of 1.57 ± 2.86 trials out of 18. Based on the frequencies at which gaze directions were associated with mental states, the k-means cluster analysis grouped the mental states into the three following groups: (1) autobiographical memory, semantic memory, and working memory; (2) lying, shyness, and selective top-down attention; (3) vigilance and bottom-up attention (see Supplementary Table 1 for details of classification). In accordance with the hypothesis, participants chose one of the three internal attentional mental states to be associated with upward gazes (straight up, up left, or up right) in 58.65% of the cases, while they chose one of the three external attentional states to be associated with gazes on the horizontal line (straight ahead, left, or right) in 68.75% of the cases. To calculate those percentages, we summed the percentage at which every mental state from the category of interest was chosen for the three gaze directions of interest (e.g., the three upward directions for internal attentional states). For example, if internal attention was the category of interest, we summed the percentage at which autobiographical memory has been chosen with the percentage of semantic memory and the percentage of working memory. Then, the summed percentages for the three gaze directions were averaged.

While the intra-individual consistency of responses for a specific mental state was moderate for straight-down gaze (*K* = 0.40) and substantial for straight-ahead gaze (*K* = 0.62) according to Cohen’s Kappa coefficients, the consistency was only fair for the other gaze directions: leftward gaze, *K* = 0.28; up-left gaze, *K* = 0.23; straight-up gaze, *K* = 0.33; up-right gaze, *K* = 0.29; rightward gaze, *K* = 0.31; down-right gaze, *K* = 0.37; down-left gaze, *K* = 0.38.

**Figure 2:**
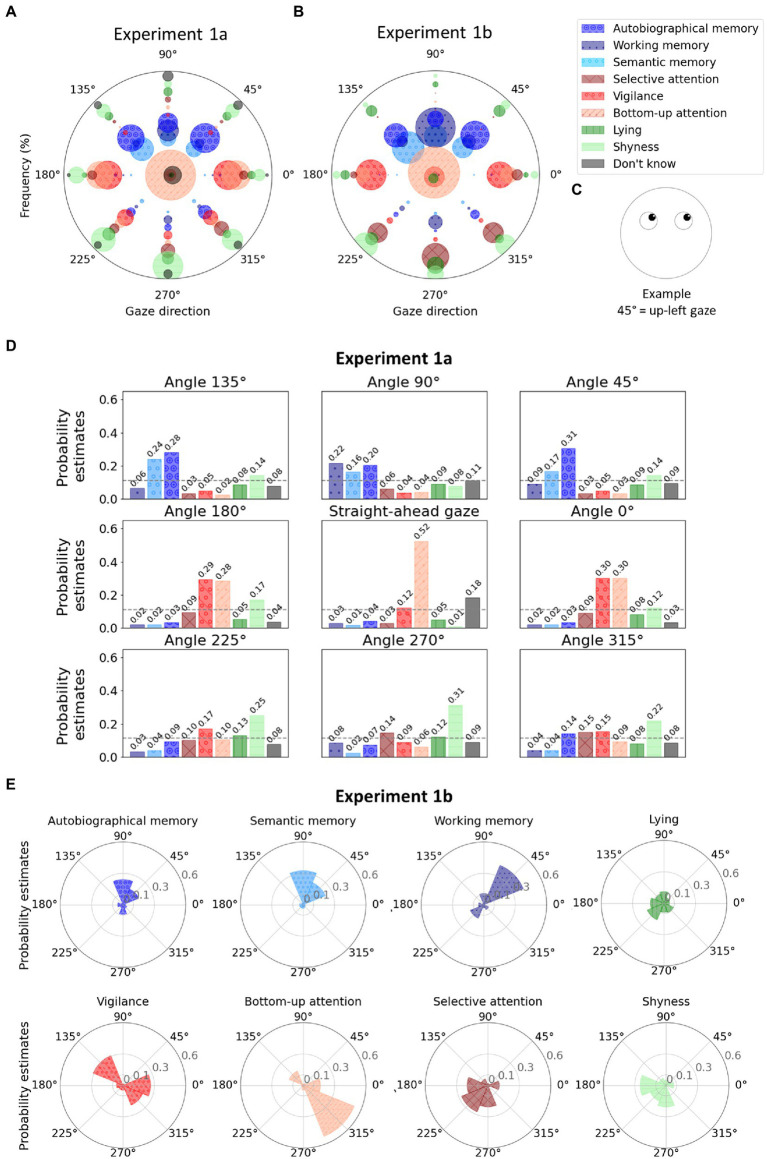
**(A)** Polar graph in which each angle represents a gaze direction. For each gaze direction, the radii of the bubbles represent the frequency at which a given mental state (see colors) was associated with the given gaze direction during Experiment 1a. The size of the bubbles depends on the frequency of the responses. Note that the position of the bubbles along the y-axis has no meaning and was chosen to avoid overlapping and allow better visualization. The bubbles in the center of the graph (coordinates 0,0) represent the answers for the question where the stimulus was presented with straight-ahead gaze. **(B)** Polar graph representing the frequencies at which gaze directions (see angles) were associated with mental states (see colors) during Experiment 1b. **(C)** Example of a stimulus, illustrating the correspondence between angles in polar coordinates and gaze directions. **(D)** For the Experiment 1a, one bar plot per gaze direction where the bars represent the probability estimates from the multinomial logistic regression for each mental state. The dashed gray lines represent chance level (i.e., 0.11). **(E)** For experiment 1b, one polar plot per mental state where the probability estimates from the multinomial logistic regression are represented for each gaze direction (chance level at 0.10). The legend shows the correspondence between colors and mental states. Shades of blue were used for internal attention, shades of red for external attention, shades of green for control proposals, and black for “Do not know” responses. Hatching has been added to make the figure color-blind friendly.

As hypothesised, results showed that the frequencies at which people associated specific mental states to faces were significantly associated with gaze directions according to the contingency Pearson’s chi-square test [*χ*^2^(56) = 787.00, *p* < 0.001]. Detailed visualization of frequencies at which every mental state was associated with every gaze direction are reported in Figure 2A. The multinomial logistic regression classification reached an overall accuracy of 0.30. The probability estimates are reported in Figure 2D. We observed that the three types of memory are all preferentially associated with upward gazes. Slight differences were however observed in the upward direction: the estimated probability of working memory to be associated with straight-up gaze was 0.22, while autobiographical and semantic retrieval were more likely to be associated with up-left (0.31 and 0.17) or up-right gazes (0.24 and 0.28 for the two types of retrieval, respectively) with a slight preference for autobiographical memory when the gaze was directed up left.

### 2.2. Experiment 1b

#### 2.2.1. Participants

Participants were the same as for Experiment 1a. Experiments 1a and 1b were run consecutively as two parts of the same experiment.

#### 2.2.2. Materials and survey

Participants were presented with the 8 written descriptions of mental states (see Table 1) and were asked to choose, for each of them, the face that corresponded best. Participants had the choice between the nine faces from Figure 1, which each showed one of the nine gaze directions described previously (see section 2.1.2). To avoid random choices, participants were always given the option of ticking “I do not know.” This experiment was composed of nine trials (one for each mental state) including the validation trial (*“This trial will allow us to make sure that you completed the survey seriously. For this trial, please choose the face number 4.”*), which were presented in randomized order. The instructions stated: “Imagine that you are doing the given mental task, which face would you associate with it?”

The instructions also emphasized that there was no right or wrong answer. Participants were encouraged to trust their first impression in choosing their answers. All the stimuli needed to reproduce the experiment are available on OSF.

#### 2.2.3. Analyses

The contingency Pearson’s chi-square test was performed similarly to the one of Experiment 1a. In case of significant results, we planned to investigate if gaze direction could be predicted from a given mental state with probabilities above chance level. We ran a multinomial logistic regression with the responses of participants (gaze direction choices) as dependent categorical variable and mental states as independent categorical variable. We used Logistic Regression from the sklearn package in Python 3.7.6. We calculated both the overall accuracy of the model and the probability estimates for every mental state. Given our interest in internal attention, we focused on the trials concerning memory states. As mentioned previously, we expected people to choose preferentially upward directions of gaze (up left, up right, or straight up).

Data visualizations provided information about which gaze directions were linked to which types of mental states and allowed us to compare those associations between Experiment 1a and Experiment 1b.

#### 2.2.4. Results

Participants abstained from answering on an average of 0.41 ± 1.21 questions out of 8. Similarly to Experiment 1a, results showed that people interpreted gaze directions as a significant cue for specific mental states according to the contingency Pearson’s chi-square test, which rejected the independence between the variables [*χ*^2^(56) = 1278.47, *p* < 0.001].

When asked to associate a gaze direction to *autobiographical memory retrieval*, 71.88% of the participants chose an upward-directed gaze (straight up, 16.88%; up right, 25%; and up left, 30%). For *semantic memory retrieval trial*, 79.38% of the participants chose an upward-directed gaze (straight up, 23.75%; up right, 33.75%; and up left, 21.88%). *Working memory* was also mostly associated with an upward-directed gaze by 66.88% of the participants (straight up, 41.88%; up right, 11.25%; and up left, 13.75%).

Figures 2A,B allow a detailed and comparable visualization of the raw frequencies at which each mental state was associated with each gaze direction both in experiments 1a and 1b. The frequency from which those visualizations were created are provided in Table 2 and Table 3 for experiments 1a and 1b, respectively. The multinomial logistic regression classification reached an overall accuracy of.33. The probability estimates are reported in Figure 2E.

**Table 2:** Frequencies of responses (in %) during Experiment 1a for the different gaze directions.

Mental state	Left	Up left	Straight up	Up right	Right	Down right	Straight down	Down left	Straight ahead
Autobiographic memory	2.81	30.93	20.62	28.43	3.12	9.37	7.18	14.37	4.06
Semantic memory	1.87	16.87	16.56	24.68	1.87	4.06	2.18	3.75	1.25
Working memory	1.56	9.06	22.18	6.56	1.56	3.12	8.43	4.06	2.81
Vigilance	30.62	4.68	3.43	4.68	29.68	17.18	8.75	15.31	12.50
Selective attention	9.06	3.12	5.93	3.12	9.37	10.00	14.68	15.00	2.50
Bottom-up attention	30.62	2.81	3.75	1.87	28.75	10.31	5.93	9.06	53.13
Lying	8.12	8.75	9.06	8.43	5.00	13.12	12.18	8.12	5.00
Shyness	12.18	14.37	7.50	14.37	17.18	25.31	31.56	21.87	0.00
Do not know	3.12	9.37	10.93	7.81	3.43	7.50	9.06	8.43	18.75

**Table 3:** Frequencies of responses (in %) during Experiment 1b for the different mental states.

Mental state	Left	Up left	Straight up	Up right	Right	Down right	Straight down	Down left	Straight ahead	Do not know
Autobiographic memory	1.88	30.00	16.88	25.00	1.25	5.00	2.50	9.37	3.75	4.37
Semantic memory	1.25	21.88	23.75	33.75	1.25	3.13	3.13	3.13	1.88	6.87
Working memory	0.00	13.75	41.88	11.25	0.63	5.00	14.27	2.50	5.00	5.62
Vigilance	27.50	1.23	0.63	0.63	31.88	6.25	4.37	4.35	21.25	1.88
Selective attention	11.25	1.25	0.63	1.25	6.87	20.63	20.63	21.25	1.25	7.50
Bottom-up attention	15.50	0.00	1.87	0.00	15.63	1.25	1.25	4.35	54.38	3.75
Lying	5.00	11.87	5.63	11.25	9.37	31.13	17.50	8.75	10.00	7.50
Shyness	7.50	9.37	3.75	5.00	7.50	25.63	16.87	21.25	0.00	3.13

### 2.3. Experiment 2

#### 2.3.1. Participants

Data were collected from July to October 2020. A total of 367 participants clicked on the link to start the study; 177 participants completed the study, but 14 gave a wrong answer to the validation trial. The results include responses from 163 participants (62.58% women; 83.44% right-handed) aged 18–50 years (*M* = 31.16 ± 8.35), with an education level between 11 and 20 years (*M* = 17.13 ± 2.35).

#### 2.3.2. Materials and survey

**Figure 3:**
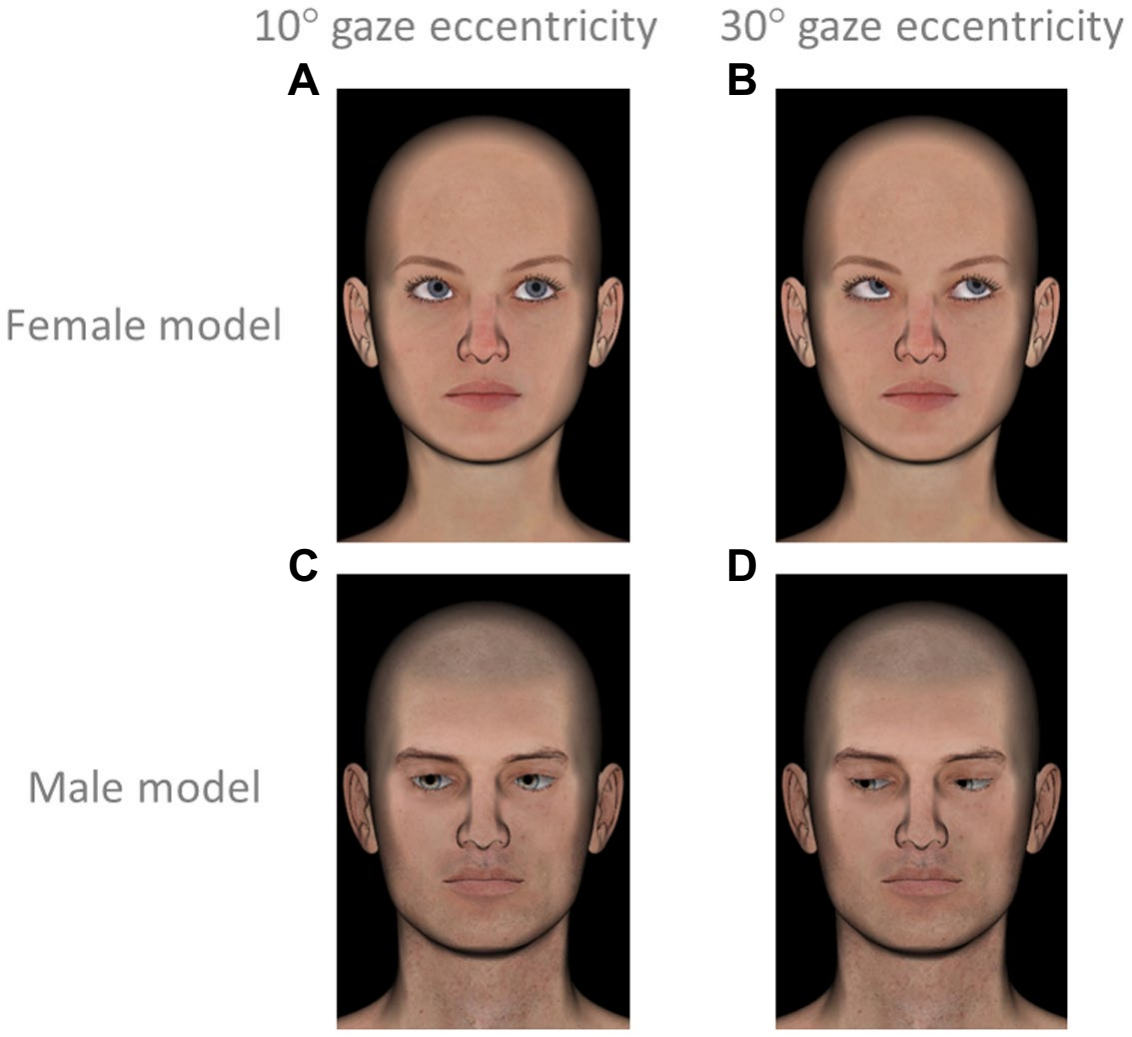
Examples of stimuli presented in Experiment 1b. **(A)** female face with gaze at low eccentricity (direction 45°, up left); **(B)** female face with gaze at high eccentricity (direction 45°, up left); **(C)** male face with gaze at low eccentricity (direction 225°, down right); **(D)** male face with gaze at high eccentricity (direction 225°, down right).

Participant were presented representations of faces in 3D created with the open-source modeling software DAZ Studio 4.12 (from).^4^ Faces differed depending on the orientation of the eyes according to nine directions expressed according to the positions of eyes relative to the face in polar coordinates (same as in Experiment 1a and 1b) and 2 levels of gaze eccentricity approximately 10° rotation (see Figures 3A,C for examples) or 30° rotation (see Figures 3B,D for examples). To avoid a confounding factor related to gender, each of the 18 gazes was presented twice, once on a female and once on a male model (see Figure 3 for models). As the study focused on gaze direction, all faces had the same pose and a neutral emotionless expression. Each face was accompanied by a multiple-choice question where participants were offered several descriptions and were asked to choose the option that best corresponded to the mental state of the face. For each face, participants had the choice between the same 8 options as in Experiments 1a and 1b (listed in Table 1). This experiment therefore included 34 trials [= (8 averted gaze directions * 2 face models * 2 levels of eccentricity) + (1 straight-ahead gaze * 2 face models)] plus one validation trial. Trials were presented one by one in randomized order. For every trial, the eight mental state options were presented in a different random order.

Instructions suggested the following to the participants: “Imagine that the stimulus is a person in front of you. How would you interpret their mental state?” and emphasized that there was no right or wrong answer. Participants were encouraged to trust their first impression in choosing their answer. To avoid random choices, participants were always given the option of ticking “I do not know.” All the stimuli needed to reproduce the experiment are available on OSF. At the end of the experiment, participants had the possibility to leave free commentaries about the experiment.

#### 2.3.3. Analyses

Raw data were exported as an Excel file (publicly available on OSF). Analyses were performed with Python 3.7.6 and TIBCO Statistica 13.5.0.17. To evaluate whether people preferentially associated certain mental states with specific gaze directions, we compared the frequencies at which participants chose a specific mental state for each gaze direction by means of Pearson’s chi-square test (χ^2^), testing the independence of variables (mental state vs. gaze direction) in a contingency table. Because this test was run separately for the two levels of gaze eccentricity, the Bonferroni correction was applied to correct α for the number of tests (α = 0.025). To test the hypothesis that internal attentional states are associated more often with upward gazes (straight up, up left, or up right) when the gaze was at far eccentricity, we performed a chi-square test comparing the frequency at which one of the three internal attentional states was chosen for the three upward-directed gazes against chance level for both low and high eccentricity separately. In case of significant results, we planned to investigate if mental states could be predicted from gaze direction with probabilities above chance level. We therefore ran a multinomial logistic regression with the responses of participants (mental state choices) as dependent categorical variable and both gaze direction of the stimuli and level of gaze eccentricity as two independent categorical variables. We calculated both the overall accuracy of the model and the probability estimates for every gaze direction. Data visualizations provided information about which gaze directions are linked to which types of mental states and allowed us to look at the differences depending on the eccentricity of the gaze (lower or higher rotation).

#### 2.3.4. Results

Participants abstained from answering on an average on 3.34 ± 3.75 trials out of 34. (Interestingly, if one excludes the straight-ahead gaze common to the two conditions, the average number of abstentions was 0.83 in the high eccentricity condition as compared to 1.69—more than twice as high—in the low eccentricity condition.) In accordance with the previous experiments, results showed that the frequencies at which people associated specific mental states to faces were significantly associated with gaze directions according to the contingency Pearson’s chi-square test, and this was true for both low [*χ*^2^(56) = 499.05, *p* < 0.001] and high gaze eccentricity [*χ*^2^(56) = 787.52, *p* < 0.001]. As expected, internal attentional states (trials where participants chose one of the three memory states) were significantly associated with an upward gaze (straight up, up left, or up right) for both low (51.53%) and high gaze eccentricity (48.57%), but contrary to our hypothesis, internal attentional states were not preferentially associated with high eccentricity compared to low eccentricity [*χ*^2^(1) = 0.35, *p* = 0.553].

**Figure 4:**
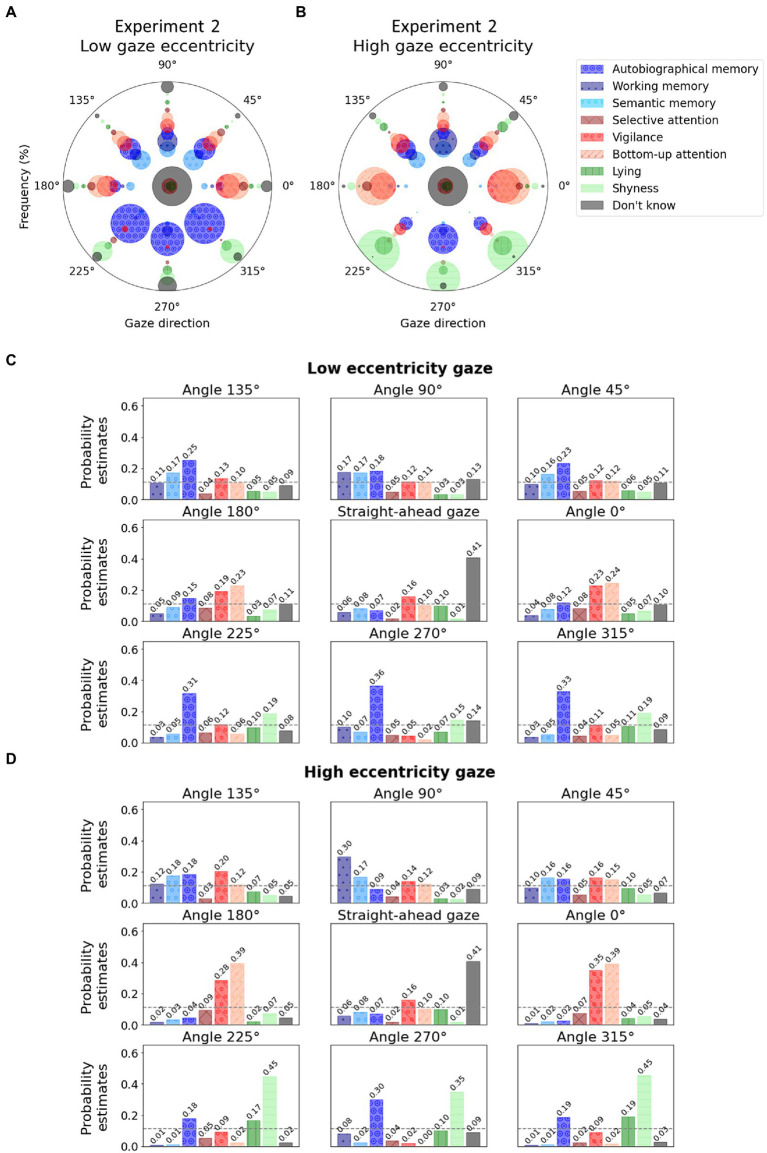
Polar graphs in which each angle represents a gaze direction. **(A)** For each gaze direction, the radii of the bubbles represent the frequency at which a given mental state (see colors) was associated with the given gaze direction during Experiment 2 for the condition with low gaze eccentricity (for examples of stimuli, see Figure 2); **(B)** For each gaze direction, the bubble radii represent the frequency at which a given mental state (see colors) was associated with the given gaze direction during Experiment 2 for the condition with high gaze eccentricity. **(C)** Low eccentricity condition. The bars represent the probability estimates from the multinomial logistic regression for each mental state. The dashed gray lines represent chance level (i.e., 0.11). **(D)** High eccentricity condition. For graph conventions and legend, see Figure 2.

Detailed visualization of raw frequencies at which each mental state was associated with gaze direction for low and high gaze eccentricities separately are reported in Figure 4 for comparison. Frequency from which those visualizations were created are available in Table 4 and Table 5 for low and high gaze eccentricity, respectively. The multinomial logistic regression classification reached an overall accuracy of 0.31. The probability estimates are reported in Figures 4C,D for low and high gaze eccentricity, respectively.

**Table 4:** Frequencies of responses (in %) during Experiment 2 for the condition with gaze at low eccentricity.

Mental state	Left	Up left	Straight up	Up right	Right	Down right	Straight down	Down left	Straight ahead
Autobiographic memory	8.28	22.39	12.88	21.78	12.58	40.49	34.97	42.02	6.44
Semantic memory	6.13	21.16	16.26	19.32	8.28	5.52	4.29	5.21	7.97
Working memory	3.37	8.59	20.55	11.66	3.37	3.37	10.43	2.76	5.52
Vigilance	24.85	15.34	14.42	15.34	21.16	6.44	3.68	3.99	15.95
Selective attention	8.89	7.67	4.60	5.21	8.28	6.13	3.37	1.84	1.53
Bottom-up attention	27.61	14.11	11.35	14.72	22.70	1.23	0.31	0.61	10.12
Lying	4.60	3.68	4.29	4.29	3.99	7.05	9.20	10.12	10.12
Shyness	4.60	1.84	2.45	3.37	6.44	20.25	14.72	24.85	1.23
Do not know	11.66	5.21	12.88	4.29	12.88	9.51	19.02	8.28	41.10

**Table 5:** Frequencies of responses (in %) during Experiment 2 for the condition with gaze at high eccentricity.

Mental state	Left	Up left	Straight up	Up right	Right	Down right	Straight down	Down left	Straight ahead
Autobiographic memory	3.99	15.95	11.66	19.94	5.21	13.50	30.67	14.11	6.44
Semantic memory	2.76	14.11	17.78	16.56	3.37	0.92	3.37	0.92	7.97
Working memory	0.92	10.43	28.22	11.66	2.15	0.61	7.97	0.92	5.52
Vigilance	34.05	14.72	12.58	19.32	27.61	11.96	2.15	12.58	15.95
Selective attention	7.05	4.29	4.29	2.15	9.51	5.21	4.29	3.37	1.53
Bottom-up attention	37.73	13.80	12.27	9.81	39.57	4.29	0.92	3.68	10.12
Lying	3.99	10.74	2.15	7.97	1.53	18.10	8.89	19.32	10.12
Shyness	6.44	6.75	2.76	5.83	7.67	44.17	35.28	42.64	1.23
Do not know	2.76	9.20	8.89	6.75	3.07	0.92	6.44	2.45	41.10

## 3. Discussion

During daily life interactions, humans constantly switch their attention between external stimulations and their internal mental world when they try to retrieve information from memory. While joint attention allow interacting individuals to pay attention to the same external object (Frischen et al., 2007), our results show that upward gaze aversion is a relevant cue to infer when the attention of others is focused on their inner mental world. In accordance with our main hypothesis, people significantly associated one of the internal attentional states with an upward gaze (up left, straight up, or up right; 58.65% of the participants on average). This pattern seems robust since results from the two experiments coincide. In contrast, external attention was mostly associated with a straight-ahead gaze or gaze remaining on the horizontal axis and directed either to the left or to the right (68.75% of the participants on average). Results also show that control mental states—i.e., lying and shyness—were related to downward gazes, which corroborate studies showing that social embarrassment increases gaze aversion (Moukheiber et al., 2010) and makes people look down (Costa et al., 2001). Based on the frequency at which participants associated a specific mental state to a gaze direction, the cluster analysis grouped the different mental states the same way that we categorized them *a priori* while designing the experiment (except for the top-down selective attention state). Overall, these results support the idea that the direction of gaze direction is a highly relevant social cue to infer in which mental state an observed person is. It also demonstrates that gaze directed upward is strongly suggestive of attention directed to the internal state, which is consistent with upward-directed gazes being interpreted as a thinking state (Baron-Cohen and Cross, 1992). An interpretation of such results is that while external attention requires focusing on the part of the space where the information lies, internal attention brings the gaze towards more empty places, relatively poor in visual distractors (Salvi and Bowden, 2016). We assume that observers during social interactions have learned from experience that the probability of finding relevant external information is significantly lower in the upper part of the space and therefore infer that the observed person is in an internal state when the gaze switches upward.

Internal attention has been associated with different types of eye movements, such as longer blink duration, higher blink frequency, or change in pupil diameter or eye vergence (e.g., Benedek et al., 2017; Walcher et al., 2017). Interestingly, a recent study has shown that eye movements related to internal attention can be modified when occurring during social interactions: facing the experimenter while retrieving memories generates shorter fixations along with longer saccades compared to facing a screen (Lenoble and El Haj, 2021). From a cognitive point of view, all the eye movements reported above participate in *perceptual decoupling* (Smallwood and Schooler, 2006)—the reduction of visual processing to optimize internal cognition. Nevertheless, it seems difficult to imagine that those eye movements are interpretable as cues of the attentional state during social interactions because they are too subtle and too variable (for example, depending on the surrounding level of light). They also take place in a rapid and very dynamic way, and require *a posteriori* averaging of their duration or frequency over a period of time. They can therefore hardly explain how people can interpret the attentional state of an observed person based on static photos (Benedek et al., 2018). In that sense, our results support that gaze aversion, another strategy of perceptual decoupling (Glenberg et al., 1998; Abeles and Yuval-greenberg, 2017), can be an efficient social cue to infer internal memory mental states.

Our results did not support our second main hypothesis, according to which high gaze eccentricity is associated with internal attention. This hypothesis was based on the fact that eye-head coordination plays a crucial role in visual processing of external stimulations—at high eccentricity, during visual external attention, the head follows the eyes to keep them in a comfortable oculomotor range and maintain an accurate visual acuity (Fang et al., 2015; Franchak et al., 2021). But during gaze aversion, where the goal is to decouple the attention from the environment, we expected high eccentricity to support perceptual decoupling. Surprisingly, our second experiment did not show any significant effect of the eccentricity of the gaze (10° or 30°) on the frequency at which upward gazes were associated with internal attention. To investigate this feature further, it would be interesting to compare mental states that people attribute to stimuli where the head follows the eyes to stimuli with a mismatch between head and gaze direction. Additionally, we would like to mention that low and high gaze eccentricity differed by the degree of eyelid closure, a potential confounding factor that we did not evaluate. Indeed, the occlusion of the eyelid varies naturally according to the eccentricity of the gaze, in particular for gazes directed downward (which explains why Figure 3C has more wide-open eyes than Figure 3D). We intentionally kept this variable to make the stimuli more realistic, but since the visibility of the sclera is supposed to be a social cue indicating the position of the eye (Kobayashi and Kohshima, 2001), this factor could have affected participants’ social interpretation ability. In addition, how wide the eyes are open could be an indicator of vigilance, adding to the importance of studying this factor in future studies.

While exploring the differences between the different internal memory states, results from Experiments 1a and 1b converged and showed that autobiographical retrieval, semantic retrieval, and working memory were all associated with upward gazes. Subtle differences were however observed, since working memory was significantly associated with straight-up gaze while autobiographical and semantic memory were preferentially associated with either up-right or up-left gazes. Unexpectedly, however, Experiment 2 shows that autobiographical memory retrieval was also highly associated with gazes directed downward (39%), at low eccentricity only. Following the hypothesis of the cognitive load theory, looking down towards the floor could also help reduce visual distractors (Doherty-Sneddon et al., 2001). This effect was only observed for autobiographical memory retrieval and not for the other internal attention states (semantic memory and working memory), which suggests differences between gaze aversions depending on the type of memory. This reveals the possible existence of different types of gaze aversions and opens new research perspectives. It would be interesting to investigate how certain cognitive processes influence not only direction but also other features of gaze aversions, such as gaze eccentricity, temporality, or duration.

Because gaze aversion is generally related to cognitive effort (Doherty-Sneddon and Phelps, 2005), the differences or similarities that we observed between the internal mental states that we used (i.e., autobiographical memory, semantic memory, and working memory) could be partially due to cognitive resource requirements. Indeed, autobiographical memory retrieval can be more or less demanding since it can sometimes “pop up” spontaneously and automatically in daily life in response to environmental cues (Berntsen, 2010), whereas at other times a strong effort is required to retrieve specific details. Working memory is, in contrast, generally cognitively demanding, while a characteristic of semantic memories is that they are more readily accessible. Based on informal observations, it appears that low eccentricity gaze aversions seem shorter and faster. Even though this claim should be empirically tested, we assume that low eccentricity gaze aversion could be related to more fluctuating memory states, while high eccentricity gaze aversion could be related to effortful memory states requiring complex cognitive processes, such as mental time travel or detailed retrieval of a scene. The observations could also be explained by the fact that high eccentricity gaze aversions would be used as an implicit, but conspicuous, signal of communication. Interestingly, the morphology of the human eye presents one of the proportionally largest areas of white sclera among all animals, which emphasizes the relevance of the sclera as a social signal since it indicates distinctly the position of the gaze (Kobayashi and Kohshima, 2001). Looking up is especially social since it makes the sclera clearly visible. Looking up at high eccentricity could signal the temporary interruption of the social interaction required to think and retrieve memories. This hypothesis is supported by a study showing that Canadian people look up while thinking only in the presence of another person, but look down when alone (McCarthy et al., 2008). At low eccentricity, looking down is quite fast and can take place in the normal course of a conversation. We assume that such gaze aversions are interpreted in a more implicit way, which is supported by the higher rate of uncertainty and “I do not know” responses in the low eccentricity compared to the high eccentricity condition. Another alternative explanation is that short downward-directed gaze aversions rather play a role in intimacy regulation during conversations (Andrist et al., 2014). The fact that autobiographical memories are personal could potentially explain why this type of memory has been more linked to downward gazes than other types of memory.

The present study is not without limitations. One limitation concerns the limited choices of mental states to which we constrained our studies. We restricted our study to memory states for the sake of time, and we did not include comparisons to other internal cognitions such as imagination or creativity, which have also been associated with gaze aversion (Salvi and Bowden, 2016). Another limitation involves the stimuli that we used. In our second experiment, we used 3D models with neutral emotion where only gaze direction varied. The objective was to study the influence of this parameter in a controlled experimental design, but this is probably far from the way gaze aversion appears in everyday life. We expect gaze aversion to be linked to other facial cues such as frown or eye divergence, which should be taken into account in future studies. Furthermore, we used static stimuli, but the dynamic of eye movement might play a crucial role in such behavior, since Benedek et al. (2018) showed that people are better at discriminating whether the attentional focus is external or internal based on video clips than based on pictures. Also, we used neutral emotionless faces, but facial expressions of emotions might also be one cue involved in the inference of mental state since episodic memories are more associated with emotional expressions than semantic ones (El Haj et al., 2016), for example. All these social cues, gaze and emotions, are dynamics and could be preferentially processed by the face network specialized in the dynamic aspects of the faces (Haxby et al., 2000). To make the stimuli more ecological, it could also be interesting to test backgrounds of different colors, such as a black background as we did in Experiment 2, but also white (neutral) or blue as the sky under the hypothesis of a phenotypic propension to look at his usually neutral part of the external world. Finally, we should also mention a limitation in the experimental design itself. In Experiment 1a, we reported that the intra-individual concordance of responses was only fair (with kappa coefficients between 0.21 and 0.40), which means that, for the same gaze direction, participants did not always choose the same mental state. We believe that this can be due to the response modality that we used. We asked participants to choose only one mental state per gaze direction, which can constrain such exploratory studies. We could imagine other designs where we ask participants to classify the different mental states from the most to the least appropriate or give a likelihood score for each mental state. We could also ask participants to write one or two words describing the mental state they would spontaneously attribute to a given gaze direction. These designs would have the advantage of being constructed without preconceived notions about the interpretation of gaze direction. Moreover, future studies should seek to clarify whether the definitions we used to describe mental states where understood in the same way by all the participants, or whether it impacted their responses. Note that given the high consistency at the group level and between the three experiments, however, the lack of such a measure did not impact the reliability of our results. We should also highlight that running experiments 1a and 1b on different samples could have allowed for even more rigorous robustness testing.

Regarding perspectives, these results could lead to the improvement of the existing tools used to assess social cognition. For example, in the famous and widely used “Reading the Mind in the Eyes Test”(Baron-Cohen et al., 2001), most of the illustrations of people thinking represent a person with an upward-averted gaze. If other gaze directions such as downward are observed during more spontaneous memory retrieval states, it would be interesting to have a test including such items for a finer evaluation. This line of work could also guide the research for a better characterization of behavioral markers of internal attention. These might be useful in various domains, especially in human factors for attention monitoring. Over the past few years, there has been a growing interest in detecting when the attention of operators switches away from their task toward their inner world (e.g., Huang et al., 2019). Gaze aversion is a potential candidate for this detection, especially if differences in gaze aversion patterns are found between different mental states requiring internally directed attention. Detecting when operators start to think about autobiographical memories (e.g., their last holiday) might help to improve safety, but only if it does not prevent operators from using internal attention when they need to remember semantic information about their job (e.g., flight plans). Another implication is the improvement of communication between humans and robots. Designers aim to give the impression that robots have an internal mental world in order to provide the illusion that they have cognitive functioning similar to that of humans (Andrist et al., 2014). So far, designers of robots still rely on the NLP to convey a specific mental state (Ruhland et al., 2015). Because NLP is not supported by scientific evidence, it seems relevant to look at what humans infer from specific gaze directions to implement this behavior in robots and improve their realism. In that sense, our study provides evidence that humans infer specific mental states from gaze aversions and suggests that gaze aversions should be considered for developing effective conversational interactions between humans and robots.

To conclude, our studies provide preliminary evidence that gaze aversion seems robustly used by people to make assumptions regarding the mental states of others, especially to identify whether their focus of attention is externally or internally oriented. However, scientific studies about gaze aversion remain rare. This is surprising given its potential implications in various domains.

## Data availability statement

The datasets presented in this study can be found in online repositories. The names of the repository/repositories and accession number(s) can be found at: https://osf.io/93n8k/?view_only=430e6c7a3ff5456e833a7076da75ee33

## Ethics statement

The studies involving human participants were reviewed and approved by Comité d’Ethique pour les Recherches de l’Université de Toulouse (agreement 2020-273). The patients/participants provided their written informed consent to participate in this study.

## Author contributions

AS, CH, and EB engaged in conceptual and methodological discussions, and contributed to the distribution of the links to recruit the participants. AS designed the surveys, analyzed the data, wrote the draft of the manuscript, and created the figures. EB and CH provided critical feedback and revisions. All authors contributed to the article and approved the submitted version.

## Funding

AS is supported by a grant from the Aeronautics-Astronautics doctoral school of Toulouse Paul Sabatier University.

## Conflict of interest

The authors declare that the research was conducted in the absence of any commercial or financial relationships that could be construed as a potential conflict of interest.

## Publisher’s note

All claims expressed in this article are solely those of the authors and do not necessarily represent those of their affiliated organizations, or those of the publisher, the editors and the reviewers. Any product that may be evaluated in this article, or claim that may be made by its manufacturer, is not guaranteed or endorsed by the publisher.

## Footnotes

^1^ https://osf.io/93n8k/?view_only=430e6c7a3ff5456e833a7076da75ee33

^4^ www.Daz3d.com
