# Supplementary material for: Gaze direction as a facial cue of memory retrieval state
Source: Front Psychol. 2022 Dec 22;13:1063228. doi: 10.3389/fpsyg.2022.1063228 (PMC9813397; doi:10.3389/fpsyg.2022.1063228)
Supplement: Supplementary file 1 [file Table_1.DOCX]

Supplementary Material

Gaze direction as a facial cue of memory retrieval state

Anaïs Servais*, Christophe Hurter, Emmanuel J. Barbeau

* Correspondence: Anaïs Servais: Anais.servais@cnrs.fr

# Supplementary Tables

Supplementary Table 1. Classification of mental states in 3 clusters (columns) depending on the frequency at which they were chosen in response to a gaze direction in experiment 1. The superscripts represent the expected classification (1: internal attention; 2: control; 3: external attention).

| Cluster 1 | Cluster 2 | Cluster 3 |
| --- | --- | --- |
| Autobiographical memory^1^ | Shyness^2^ | Vigilance^3^ |
| Semantic memory^1^ | Lying^2^ | Bottom-up attention^3^ |
| Working memory^1^ | Selective top-down attention^3^ |  |

# Supplementary Figures


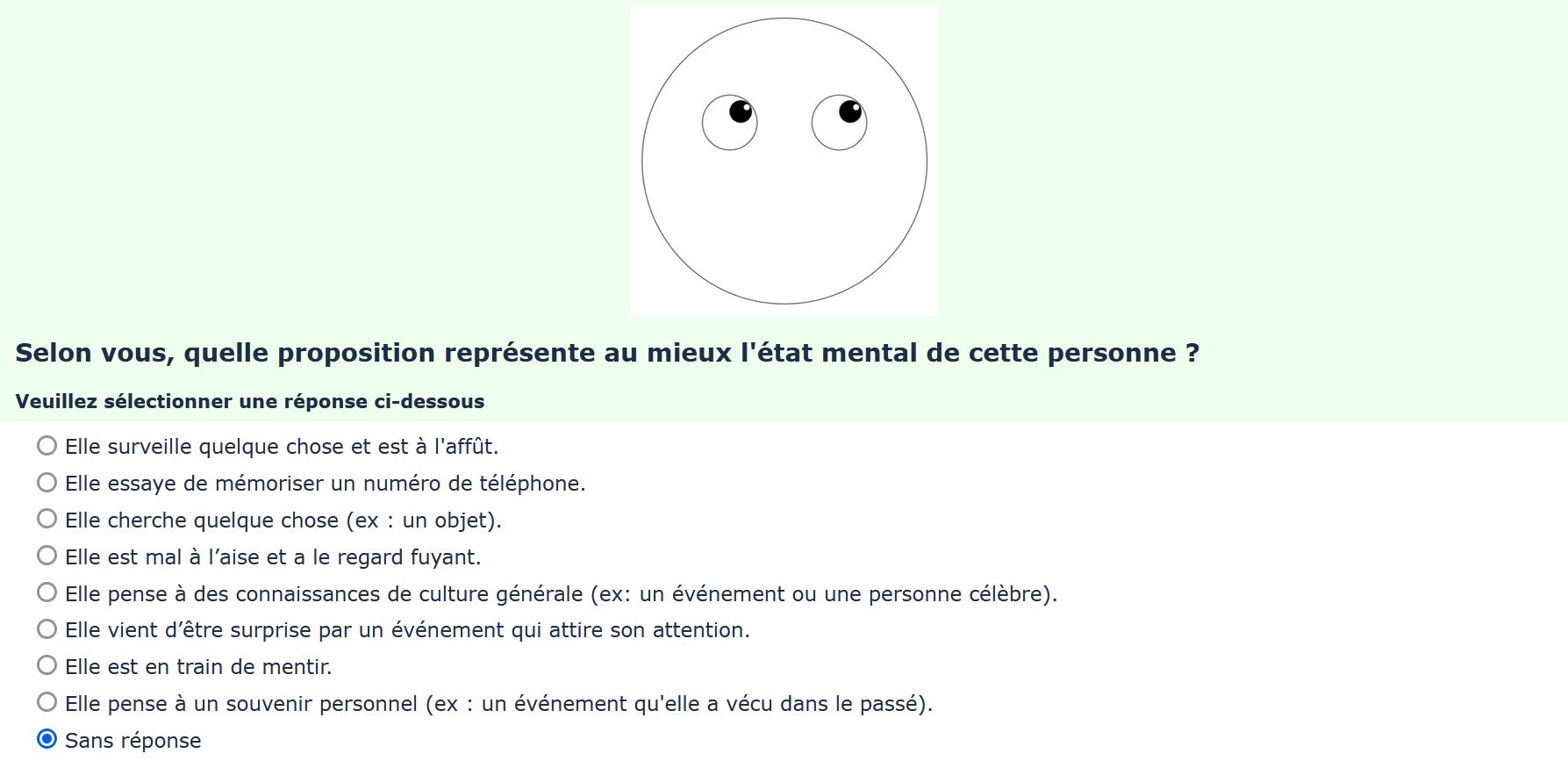


**Supplementary Figure 1.** Screenshot of a trial as displayed for the participants. English translation below.

“*Which statement do you think represents best this person’s mental state?*

*Please select one of the answers below*

- *The person is watching something while being on the lookout.*
- *The person is trying to memorize a phone number.*
- *The person is looking for something (e.g., an object).*
- *The person is being uncomfortable and avoiding eye contact.*
- *The person is thinking of general knowledge (e.g., famous event or person).*
- *The person is being surprised by an event that catches attention.*
- *The person is lying.*
- *The person is thinking of personal memories (e.g., an event lived in the past).*
- *No answer*”
